# Consistency and precision of cancer reporting in a multiwave national panel survey

**DOI:** 10.1186/1478-7954-8-20

**Published:** 2010-06-25

**Authors:** Anna Zajacova, Jennifer Beam Dowd, Robert F Schoeni, Robert B Wallace

**Affiliations:** 1 Department of Sociology, University of Wyoming, Laramie, WY, USA; 2 School of Public Health, Hunter College, City University of New York (CUNY), New York, NY, USA; 3 CUNY Institute for Demographic Research, CUNY, New York, NY, USA; 4 Institute for Social Research and Ford School of Public Policy, University of Michigan, Ann Arbor, MI, USA; 5 Department of Epidemiology, University of Iowa, Iowa City, IA, USA

## Abstract

**Background:**

Many epidemiological studies rely on self-reported information, the accuracy of which is critical for unbiased estimates of population health. Previously, accuracy has been analyzed by comparing self-reports to other sources, such as cancer registries. Cancer is believed to be a well-reported condition. This paper uses novel panel data to test the consistency of cancer reports for respondents with repeated self-reports.

**Methods:**

Data come from 978 adults who reported having been diagnosed with cancer in at least one of four waves of the Panel Study of Income Dynamics, 1999-2005. Consistency of cancer occurrence reports and precision of timing of onset were studied as a function of individual and cancer-related characteristics using logistic and ordered logistic models.

**Results:**

Almost 30% of respondents gave inconsistent cancer reports, meaning they said they never had cancer after having said they did have cancer in a previous interview; 50% reported the year of diagnosis with a discrepancy of two or more years. More recent cancers were reported with a higher consistency and timing precision; cervical cancer was reported more inaccurately than other cancer types. Demographic and socio-economic factors were only weak predictors of reporting quality.

**Conclusions:**

Results suggest that retrospective reports of cancer contain significant measurement error. The errors, however, are fairly random across different social groups, meaning that the results based on the data are not systematically biased by socio-economic factors. Even for health events as salient as cancer, researchers should exercise caution about the presumed accuracy of self-reports, especially if the timing of diagnosis is an important covariate.

## Background

Epidemiological studies often rely on self-reported information from population surveys. These data are used to calculate the incidence and prevalence rates of various health conditions in the population, to analyze their trends over time, to study their demographic, socio-economic, health-behavioral, and clinical correlates, and to inform health policy development and evaluation. The accuracy of reports is critical for unbiased and precise estimates of population health status. Numerous studies have evaluated the accuracy of self-reported health conditions by comparing them to other sources of information, such as medical records or, in the case of cancer, cancer registries. These studies have considered a single report per individual but did not address the reliability of individual self-reports over time. This paper examines the consistency of repeated reports of cancer occurrence and precision in the reported year of cancer diagnosis.

Self-reports of health conditions often do not closely match "gold standard" information from medical records or medical examinations [1-5]. The accuracy of self-reports has been found to depend more on the type and severity of the health condition than on the respondent's demographic and socio-economic characteristics [4,6-9]. To some degree, younger, female, and more educated respondents provide more accurate reporting [6,10,11]. Almost always, more serious illnesses such as cancer are reported with higher accuracy than nonfatal chronic conditions such as hypertension [1,6,12].

As a life-threatening illness, cancer is considered a highly salient health condition that respondents are believed to recall accurately [11]. The assumption of accurate reporting may be one reason why the quality of cancer reports has attracted relatively few validation studies [13]. Some researchers have found retrospective self-reports of cancer occurrence to be reliable [10] when compared to cancer registry data, while others have identified considerable underreporting [9,13-15]. As with other conditions, respondents' socio-economic and demographic characteristics were only weakly associated with reporting accuracy; a key correlate of the quality of cancer reporting was the primary tumor site [2,11,13,14,16].

Health misreporting may be reflected not only in comparison with administrative records, but also in the consistency with which respondents report the occurrence and time of onset for a given condition over multiple interviews. The consistency of repeated retrospective reports has been examined for other outcomes, such as the timing of first intercourse [17,18], but, to our knowledge, only one previous study has analyzed the reliability and consistency of reports of chronic diseases over time [6]. Using two waves of data collected from elderly respondents in the U.S. and Taiwan, the authors analyzed the probability that respondents acknowledged a health condition during the second wave of interviews if they mentioned it at the baseline interview. They found that consistency varied across conditions, with more severe illnesses associated with a higher probability of being recalled the second time.

We focus on the reliability of repeated reporting of cancer as a salient health condition that respondents are unlikely to underreport due to forgetting. Moreover, the diagnosis tends to be a one-time event associated with results of medical tests or a doctor's diagnosis, in contrast to conditions such as diabetes, arthritis, or hypertension, where the gradual accumulation of symptoms leading to a diagnosis may complicate the recall of onset. The analysis will address two factors related to reliability: 1) inconsistency, defined as reporting "no cancer ever"' after having reported cancer in a previous wave, and 2) imprecision, defined as variation in repeated reports of the year of diagnosis. Perfectly reported data would show no instances of retrospective cancer information changing from positive to negative, and no discrepancy in the timing of onset over multiple interviews. The results will provide a new perspective on the degree of measurement error in cross-sectional surveys that collect retrospective information about cancer occurrence, and assess whether such errors occur systematically by individual-level demographic or socio-economic factors, or by disease-specific characteristics such as cancer site, degree of limitations due to cancer, or time since diagnosis.

## Methods

### Data

Analyses are based on data from the Panel Study of Income Dynamics (PSID), conducted by the Survey Research Center at the University of Michigan. The PSID began in 1968 as a longitudinal study of a representative sample of US individuals and families. Starting with a national sample of 18,230 people living in 4,800 families in 1968, the PSID has re-interviewed individuals from those families every year (biennially starting in 1997). In addition, all people born to or adopted by PSID sample members become sample numbers themselves and are followed in subsequent waves. Currently, the sample includes more than 9,000 families. Annual response rates have been 96% to 98% for the core PSID families in almost every wave, and 50% to 65% of individuals who did not respond to the study at some point returned to the study in a subsequent wave. Information is collected in telephone interviews lasting about 75 minutes. In 1999, PSID added a battery of questions about selected health conditions, including cancer, to the core items collected at every wave. Details of the survey design have been published elsewhere [19]. All data and documentation are available at http://psidonline.isr.umich.edu/. Data quality reports [20-23] are available as well, including evaluation of the self-reported health information that shows low item nonresponse and close alignment of estimates of smoking, health insurance coverage, obesity, and chronic conditions in comparison to the National Health Interview Survey [21,24,25]. The nonresponse rate for cancer questions was less than 1% from 1999 to 2003.

### Sample

Questions regarding health conditions were asked with respect to the two primary adults heading each family unit, referred to as "heads" and "wives." The analysis sample thus included all heads and wives aged 18 and above who reported having had cancer at least once during the four interview waves from 1999 to 2005.

### Measures

#### Cancer occurrence and timing

In 1999, 2001, and 2003, respondents were asked: "Has a doctor ever told you that you have or had cancer or a malignant tumor, excluding skin cancer?" In 2005, the question was phrased as: "Has a doctor ever told you that you have or had cancer or a malignant tumor?" A positive response was followed by a short series of related questions, including the time of onset. In 1999, 2001, and 2003, respondents were asked: "How long have you had this condition?" In 2005, the question was changed to: "How old were you when you were first diagnosed with cancer?" One person was interviewed in each family, and that person reported his or her own cancer information as well as cancer data for a spouse if married. We studied the sensitivity of the findings to the changes in the wording of the cancer questions in 2005. The conclusions of the paper were not affected; details are below.

#### Reporting inconsistency and timing precision

The unit of analysis was an individual, with the multiple reports of cancer occurrence categorized as follows. Inconsistency in the individuals' sequence of cancer reports was defined as a binary variable coded 1 if respondents reported in 2001, 2003, or 2005 that they had never had cancer after reporting in any previous wave that they had ever had cancer, and 0 otherwise. Timing imprecision was defined in terms of variation among the repeated reports of the year when the cancer was first diagnosed. We operationalized imprecision as the absolute difference between the maximum and minimum reported year of diagnosis. The variable was used as an ordered categorical covariate, coded as 0, 1, 2-3, and 4+ years of difference.

Consistency analyses excluded 38 individuals who completed only one interview and another 38 who completed more interviews but had only one valid data point for the cancer occurrence question. Respondents with only one cancer report were excluded because consistency was undefined for them. For the same reason, the timing precision analyses excluded an additional 398 individuals who had fewer than two responses to the time of cancer diagnosis. Of these, 22 were missing information on the year of diagnosis; the remaining 376 reported having had cancer at only one interview wave and consequently had only one timing report. For instance, an individual who reported never having cancer in 1999, 2001, and 2003, and then reported having cancer in 2005 with a year of diagnosis would be included in the consistency analyses but not in the timing precision analyses.

#### Additional cancer-related variables

Two additional characteristics related to the cancer diagnosis were used in the analyses: activity limitations due to cancer and primary cancer site. At all four waves from 1999 to 2005, the follow-up after a respondent's positive cancer report included the question: "How much does this condition limit your normal daily activities?" The 4-point scale ranged from "not at all" to "a lot." We coded the variable as 1 if respondent mentioned "a lot" or "somewhat" degree of limitations at any wave and 0 otherwise. In 2005, the survey asked for the first time about the type of tumor: "What type of cancer (do/did) you have? In what part of your body [is/was] it?" The cancer sites included breast, colon, lung, lymphoma or leukemia, melanoma, prostate, skin (not further specified), uterine, ovarian, cervical, and other. Respondents could report one or more cancer sites; however, more than 99.5% of cancer survivors reported only one type of cancer. For the 20 individuals who mentioned a second cancer site, we used the first-mentioned type.

#### Socio-demographic variables

Year of birth was calculated from age at the time of interview. Gender was coded 0 = male, 1 = female. Race/ethnicity was coded 0 = non-Hispanic white, 1 = black, Hispanic, and other. Education was reported as the highest year of schooling completed, with a range from 1 to 17 years. Marital status was defined as married or cohabiting = 0, and not married = 1 if respondents were divorced, widowed, or single at any wave. Rural/urban residence (urban as reference) was dichotomous, coded as rural if the respondent resided in a small nonmetropolitan area or a rural area at any wave. Census region was based on 1999 information and categorized as Northeast (reference), Midwest, South, and West. Finally, reports by proxies have previously been found less reliable than self-reports [24,26]. For each respondent, we generated a "self/proxy" variable, coded as "only self" if all available data were self-reported, "only proxy" if all data were reported by proxies, and "both self and proxy reports" for all other cases.

### Statistical Analysis

We first described the distribution of the individual-level and cancer-specific characteristics of the cancer survivors. We also described the distributions of key variables separately for consistent and inconsistent reporters, and tested for statistical significance of the difference between the two groups with respect to all covariates. Next, we compared the prevalence of cancer in the PSID against the Surveillance, Epidemiology and End Results (SEER) data [27]. The SEER program aggregates information from population-based cancer registries covering about 26% of the US population and is considered the most reliable source of cancer incidence and survival in the US [28]. The PSID all-site cancer prevalence rates were calculated separately for each wave using wave-specific sampling weights to obtain nationally representative estimates.

The multivariate analyses comprised two steps. First, we estimated a series of nested logistic models of inconsistent reporting to evaluate characteristics associated with inconsistency in a multivariate framework. Second, we fitted a series of nested ordered logistic regression models of the timing inaccuracy (disparity between the maximum and minimum diagnosis year reported across waves), adjusting gradually for all individual-level and cancer-related covariates. We also showed the timing accuracy in a scatterplot matrix to help visualize the patterns of reporting of diagnosis years across the four interview waves.

We conducted an extensive set of diagnostic and sensitivity tests. We examined how the change in the wording of the cancer occurrence and timing questions in 2005 affected the responses. For the cancer occurrence, we expected that when respondents were not instructed to "exclude skin cancer," may respondents would report skin cancers for the first time. This prediction was supported by the data: 92% of respondents who indicated in 2005 that they had "skin cancer (not further specified)" (N = 88) reported no cancer previously. Exclusion of these respondents had no substantive effect on the findings. The change in the wording of questions related to the timing of cancer, from "how long have you had this condition?" to "how old were you when you were diagnosed?" also did not systematically affect the findings, as measured by correlations in the timing responses involving the year 2005, as compared to correlations including only the years 1999 to 2003. The Brant test of proportionality for the ordered logistic model of timing accuracy was not significant for the global test, as well as with respect to the single predictors except nonmelanoma skin cancer type, indicating that the proportional odds assumption was not seriously violated [29]. We also studied the impact of selecting different variable and model specifications, i.e., using age at diagnosis instead of year of diagnosis and excluding potentially influential observations such as long-term survivors. Finally, re-estimating the analyses with weights that correct for most departures from representativeness led to the same substantive conclusions as unweighted results (shown). These tests showed that the key findings were robust to different model specifications. Some coefficients were less stable; we point these out in the results section. The results of all sensitivity analyses are available on request.

## Results

Table 1 shows univariate and bivariate distributions of survivor characteristics and their cancer reports. Most respondents (72%) had cancer-occurrence reports in all four waves. The modal cancer site was breast cancer (20%), followed by nonmelanoma skin cancers (14%) and prostate cancer (11%). About 28% of the respondents reported limitations due to cancer in at least one wave. The average survivor was born in 1946 and was diagnosed in 1995, at 49 years of age.

**Table 1:** Characteristics of the Cancer Survivor Sample

			**Consistent vs. inconsistent response patterns**^**1**^				
			
	All cancer reports		**Consisten**t		Inconsistent		Difference
N	978	100%	640	71.0%	262	29.0%	
# waves reported (non-missing)							**
1	76	7.8%	N/A		N/A		
2	78	8.0%	67	10.5%	11	4.2%	
3	118	12.1%	78	12.2%	40	15.3%	
4	706	72.2%	495	77.3%	211	80.5%	
Respondent^2^							n.s.
Only self	497	50.8%	328	51.2%	146	55.7%	
Only proxy	287	29.4%	187	29.2%	64	24.4%	
Both self and proxy	194	19.8%	125	19.5%	52	19.8%	
Primary cancer site (N = 628)^3^							*
Breast	127	20.2%	116	21.0%	10	18.9%	
Colon	45	7.2%	43	7.8%	0	0.0%	
Lung	18	2.9%	16	2.9%	1	1.9%	
Lymphoma	20	3.2%	15	2.7%	2	3.8%	
Melanoma	31	4.9%	27	4.9%	4	7.5%	
Prostate	69	11.0%	65	11.8%	4	7.6%	
Nonmelanoma skin	88	14.0%	83	15.0%	4	7.5%	
Uterine	32	5.1%	28	5.0%	4	7.6%	
Ovarian	15	2.4%	13	2.4%	1	1.9%	
Cervical	50	8.0%	34	6.2%	10	18.9%	
Other/DK/NA	133	22.2%	112	20.3%	13	24.5%	
Limitations due to cancer							***
Some or a lot at any wave	272	27.8%	198	30.9%	48	18.3%	
Timing of diagnosis (N = 964)							
Mean year of diagnosis^4^	1995.3	(8.7)	1996.0	(8.9)	1993.3	(8.6)	***
Mean age at diagnosis^4^	49.4	(18.2)	52.1	(16.9)	43.1	(18.6)	***
Reports of diagnosis year^5 ^(N = 504)							n.s.
No discrepancy	101	20.0%	80	20.6%	21	18.1%	
Discrepancy of 1 year	151	30.0%	121	31.2%	30	25.9%	
Discrepancy of 2-3 years	119	23.6%	89	22.9%	30	25.9%	
Discrepancy of 4+ years	133	26.4%	98	25.3%	35	30.2%	
Survivors' characteristics							
Female	620	63.4%	386	60.3%	187	71.4%	**
Non-white	265	27.1%	153	23.9%	91	34.7%	**
Not married^6^	466	47.7%	279	43.6%	125	47.7%	n.s.
Rural residence^6^	152	15.5%	106	16.6%	35	13.4%	n.s.
Education^7^	12.7	(2.9)	12.9	(2.9)	12.4	(2.8)	**
Year of birth^7^	1945.8	(18.0)	1943.7	(16.7)	1950.1	(18.5)	***

* p < .05, **p < .01 ***p < .001

*Note: *The difference between consistent and inconsistent reports was evaluated using chi-squared tests and t-tests.

1 In/consistency is defined only for respondents with two or more nonmissing cancer reports.

2 All categories can include missing reports for some waves - i.e., 'only self' includes self or missing, but no proxy reports.

3 Collected only in 2005.

4 Calculated as the mean of all nonmissing timing reports.

5 Discrepancy is defined only for respondents with 2+ reports of diagnosis year. This constraint excludes 386 individuals who reported cancer occurrence more than once but the timing of diagnosis only once.

6 Defined as not married, rural, and south if respondents reported those categories at any of the 4 waves.

7 Defined as the mean of multiple reports per individual. There was little variation across waves in the reports.

Almost 30% of cancer survivors provided inconsistent reports, defined as reporting no cancer ever after having previously reported cancer. In bivariate tests, several covariates were significantly associated with inconsistent reporting: a higher total number of reports, no limitations due to cancer, a diagnosis occurring a longer time ago or at a younger age, as well as being female, nonwhite, less educated, and younger. There was a statistically significant difference among the cancer types in the likelihood of inconsistent reporting (chi square = 20.1, d.f. = 11, p < .04). All 43 individuals with colon cancer provided consistent reports; for all other sites, consistency was above 86% except cervical cancer, where only 77% reports were consistent. The discrepancy between these high site-specific consistency proportions and the overall consistency of only 70% is due to the high level of inconsistency (91%) among the 297 survivors who did not report a cancer type, primarily comprising nonparticipants in the 2005 interview, when this information was collected.

Because the PSID has not previously been used to study cancer, the next step was to compare its rates against those from more established data sources, the National Health Interview Surveys (NHIS) [30] and the SEER [27]. The comparison of PSID with self-reported NHIS data was published previously; the prevalence rates from the two sources were found comparable [21]. We added a comparison against the registry-based SEER, shown in Table 2. The table compares the all-site age-standardized cancer prevalence for each of the four PSID waves against the SEER cancer data. The prevalence proportions show PSID results as comparable to the SEER estimates. The prevalence rates increased over time in PSID, although the rates were weighted to represent the year-specific US population, so we would expect little change over time. The cause of these increasing cancer rates is not entirely obvious, although the pattern has been observed in previous data-quality analyses [21].

**Table 2:** Comparison of Cancer Prevalence, PSID versus SEER

	Prevalence (per 1,000)
SEER, 2006	38.6
PSID, 1999	36.9
PSID, 2001	43.4
PSID, 2003	44.6
PSID, 2005	44.7

*Note*: The data are age-standardized using U.S. population age 20 and above. Prevalence is defined as 16-year limited duration all-site cancer prevalence, the longest-duration prevalence published in SEER. The PSID rates are based on wave-specific reports about the past occurrence of cancer; all individuals who report having had cancer in the past 16 years are counted in the numerator of the rates. Respondents reporting nonmelanoma skin cancers are not included in the numerator in order to match SEER data, which do not include NM skin cancers. The PSID data are weighted to represent the population. The detailed age-specific prevalence rates are not shown for parsimony but are available on request.

Table 3 builds on the bivariate analyses in Table 1, assessing the effect of covariates on inconsistent reporting in a multivariate framework using nested logistic models. In general, individual-level characteristics were not systematically related to a higher likelihood of inconsistency; there was some suggestion that younger and less educated adults reported cancer less inconsistently (models 1 and 2), but model 3 indicated that these tendencies may be related instead to the time of onset or cancer type. Women seemed somewhat more likely to provide inconsistent reports: net of all other characteristics in model 3, women were almost 2.5 times more likely to have inconsistent reports than men (p = 0.056; in weighted models OR = 4.4 and p = 0.02). The strongest predictor of inconsistent reports was the diagnosis year: more recent cancers were reported with a significantly higher consistency (OR = 0.95, p < .001). The results also suggest that adults who provided fewer reports were more consistent; however, we consider this finding less robust because it was not significant in weighted models (available on request). Finally, cancer type had some effect on inconsistency: compared to breast cancer, cervical cancer was reported less consistently across all model specifications. We note that several point estimates are substantively large but not statistically significant due to the relatively small sample size; for instance, there were only two inconsistent cases with a lymphoma.

**Table 3:** Correlates of Inconsistent Reporting (OR, 95% CI).

	Model 1		Model 2		Model 3	
Year of birth	1.02***	1.01,1.03	1.02***	1.01,1.03	1.01	0.99,1.04
Female	1.31	0.94,1.83	1.24	0.88,1.74	2.47	0.98,6.25
Nonwhite	1.29	0.91,1.82	1.29	0.91,1.84	0.98	0.46,2.08
Not married	1.07	0.79,1.46	1.19	0.84,1.69	1.09	0.52,2.32
Education	0.92**	0.86,0.97	0.91**	0.86,0.97	1.07	0.94,1.22
Rural	0.71	0.46,1.11	0.72	0.46,1.12	0.40	0.14,1.11
Midwest (NE = ref.)	1.56	0.96,2.51	1.57	0.97,2.55	1.62	0.58,4.53
South	1.19	0.74,1.91	1.22	0.76,1.96	2.39	0.92,6.21
West	1.18	0.70,2.00	1.24	0.73,2.11	1.39	0.47,4.14
Number of reports			1.58**	1.12,2.24	3.51*	1.10,11.19
Respondent (self = ref.)						
Only proxy			0.94	0.61,1.43	1.07	0.46,2.53
Self and proxy			1.17	0.75,1.81	1.83	0.77,4.34
Diagnosis year					0.95***	0.92,0.98
Cancer site (Breast = ref.)						
Lung					1.98	0.21,18.86
Lymphoma					2.92	0.51,16.80
Melanoma					2.10	0.55,8.08
Prostate					2.53	0.54,11.74
Skin					0.58	0.16,2.12
Uterine					1.37	0.37,4.99
Ovarian					0.97	0.11,8.86
Cervical					3.30*	1.07,10.14
Other					2.24	0.82,6.15
Limitations					0.84	0.37,1.88
N	902		902		562	

* p < .05, ** p < .01 *** p < .001

Exponentiated coefficients; 95% confidence intervals in second column. Unweighted results.

Finally, Table 4 presents findings on correlates of imprecise reporting of the diagnosis year. In contrast to inconsistency, which seemed fairly random (uncorrelated with most predictors), there were stronger systematic patterns in timing accuracy. Two individual-level characteristics were significantly associated with imprecise reports of cancer onset: older age and less education. All reporting variables were strongly and consistently associated with inaccurate reports: inconsistency of cancer-occurrence reports, more interviews, and proxy reports. The effects of these three reporting variables were highly significant (in all cases, p < .01), substantially large, and robust across different model specifications. Two cancer-related variables predicted a greater variation in reported times of onset: older diagnosis and cervical cancer. It is of note that these two covariates were also related to inconsistent reporting.

**Table 4:** Correlates of Imprecise Reporting of the Timing of Diagnosis (OR, 95% CI).

	Model 1		Model 2		Model 3	
Year of birth	0.98***	0.97,0.99	0.98***	0.97,0.99	0.98**	0.97,1.00
Female	1.34*	0.95,1.89	1.31	0.93,1.86	1.15	0.64,2.07
Nonwhite	1.62**	1.10,2.39	1.75***	1.18,2.61	1.47	0.91,2.38
Not married	0.80	0.57,1.12	0.90	0.62,1.31	0.71	0.43,1.16
Education	0.94*	0.88,1.00	0.94*	0.88,1.01	0.90**	0.83,0.98
Rural	1.29	0.80,2.07	1.35	0.83,2.20	1.67*	0.93,2.97
Midwest (NE = ref.)	0.85	0.52,1.38	0.89	0.54,1.45	0.74	0.41,1.34
South	0.72	0.45,1.15	0.81	0.50,1.32	0.85	0.48,1.52
West	0.66	0.39,1.12	0.77	0.45,1.31	0.74	0.39,1.39
Inconsistent reports			2.42***	1.59,3.69	2.72***	1.46,5.10
Number of reports			2.20***	1.76,2.75	1.91***	1.44,2.53
Respondent (self = ref.)						
Only proxy			1.87***	1.21,2.90	1.74**	1.03,2.93
Self and proxy			2.07***	1.38,3.09	1.79**	1.07,2.99
Diagnosis year					0.94***	0.91,0.97
Cancer site (Breast = ref.)						
Colon					1.91	0.86,4.26
Lung					1.46	0.42,5.00
Lymphoma					0.78	0.26,2.39
Melanoma					1.84	0.63,5.38
Prostate					1.80	0.77,4.19
Skin					0.76	0.12,4.92
Uterine					1.78	0.72,4.37
Ovarian					1.50	0.39,5.82
Cervical					2.38**	1.02,5.58
Other					1.49	0.79,2.81
Limitations					1.34	0.87,2.07
N	504		504		384	

* p < .05, ** p < .01 *** p < .001

Exponentiated coefficients; 95% confidence intervals in second column. Unweighted results.

**Figure 1:**
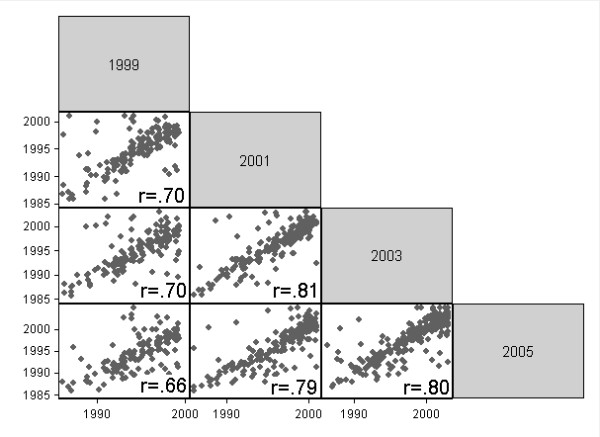
**Correlations of diagnosis years, reported across waves **Note: Includes only reports with diagnosis year > 1985 at any waves.

Figure 1 offers a picture of the variation in the reports of cancer onset across the four interviews. The correlations among pairs of reports range from r = .66 to .81. The figure shows that the scatter around the diagonals, which represent perfect timing accuracy across waves, is relatively random - there is little tendency toward systematic misreporting of the year of diagnosis. Importantly, the figure also shows that the change in the wording of the timing question in 2005 from "time since diagnosis" to "age at diagnosis" had little effect on the timing reports: There is no obvious difference between the scatterplots involving years 1999 to 2003 and the three scatterplots involving the year 2005. Interestingly, if we correlated the reported age at diagnosis, calculated from the year of diagnosis and year of birth and thus perfectly collinear with the two variables, the correlations across pairs of reports would be higher, ranging from r = .94 to .97 - simply as a function of a larger variance in ages at diagnosis, compared to diagnosis years.

## Discussion

This study analyzed the consistency and timing precision in repeated retrospective self-reports of a cancer diagnosis over four interview waves, using the Panel Study of Income Dynamics. We found that given more than one occasion to report cancer, almost 30% of respondents are inconsistent, reporting not ever having had cancer after reporting that they have had cancer at a previous interview. About 50% report the year of onset with a discrepancy of two or more years.

The consistency of cancer reports may be influenced by two opposing tendencies. On one hand, cancer is a serious, life-threatening disease. The severity and salience of the illness may increase the likelihood that respondents will report cancer with high consistency and precision [1,31]. On the other hand, cancer remains associated with stigma and discrimination [32,33], and respondents may be unwilling to report cancer during an interview, leading to underreporting or inconsistent reporting [2]. Respondents might also change their cancer report from positive to negative if they consider themselves cured or because recall issues increase with time since diagnosis. This pattern would lead to underreporting of cancer. In contrast, respondents may initially misunderstand a positive screening test or a diagnosis of precancerous lesion as evidence of cancer, and later realize that these medical findings do not constitute a cancer diagnosis [11]. Overall, little is known about patients' knowledge and understanding of their diagnoses, as well as their willingness or ability to report them accurately.

Few respondent characteristics are systematically associated with inconsistent reporting. Long-term survivors tend to be more inconsistent, perhaps because of problems with recall or lower salience of the cancer to their lives. This finding is in agreement with general survey methods literature, which shows that the accuracy of reporting of salient events declines over time [34], as well as with validity studies that find a comparable pattern specifically with respect to health events [15,16]. Our findings also corroborate studies of cancer-data validity in that individual-level characteristics had little influence on inconsistency.

The findings differ from previous studies in the importance of cancer site as a key predictor of reliable reporting: while previous studies found the primary cancer site as the key determinant of under- or overreporting [2,11,13,14,16], in our models, the cancer site was not a strong predictor of either consistency or timing precision over time, with the exception of cervical cancer, which tends to be reported relatively inaccurately. This cancer type may be particularly likely to be misreported if respondents are reporting precancerous lesions as cancer, and later correcting their cancer occurrence report. The directions of the effects of cancer sites in general, however, were consistent with the previous studies, whereby colon and breast cancers were reported with high accuracy.

Inconsistent reporters tended to also be imprecise in the timing of diagnosis, suggesting that both outcomes may be a function of the same underlying reporting tendencies. Moreover, while proxy respondents were equivalent to self-respondents in terms of reporting cancer occurrence, they were significantly less accurate in recalling the time of onset. This difference implies that if the timing of onset for health conditions is important, researchers should consider including only self-respondents. While the current analyses contribute new knowledge to our understanding of reporting of health conditions over time, there are limitations. Our analysis only addressed response consistency over time, not whether people accurately classify themselves as cancer survivors or not - the latter approach would require linking the PSID data to medical records or cancer registries. In other words, we could not distinguish between false and true positives or negatives. Given that some patterns categorized as consistent under our definition may have been in fact inaccurate (such as a report of a person who was diagnosed with cancer prior to the first interview wave but reported no cancer in the first one or more waves, only later changing their report to positive), it is startling that 30% of respondents fell into our narrowly defined inconsistent category.

The multiwave design of the PSID cancer reports presents a rich but complex data structure that warrants additional research. The current analysis, for instance, did not deal with logical inconsistencies resulting from the combination of cancer occurrence and timing reports. For instance, 186 respondents reported no cancer in 1999, 2001, and 2003, and reported having had cancer for the first time in 2005. In this group, however, 44% indicated the year of diagnosis prior to 2003 - which is incongruent with reporting no cancer during that wave. Additional analyses will be necessary to better understand such patterns of misreporting.

## Conclusions

Collecting cancer information in national population surveys that also include detailed information on employment, income, wealth, marriage, child development, and a variety of other social and economic outcomes allows the assessment of the impact of cancer on many aspects of life that cannot be examined with cancer registry data by themselves. Linking registry data to survey data would be valuable, and some national surveys are exploring this option. In the meantime, most national surveys with health data rely on self-reports of cancer diagnosis, and it is important to assess the quality of these data, which is the objective of our study.

Our results suggest that a single cross-sectional retrospective report of health conditions may contain significant measurement error, whether as a false positive or negative, in the form of not reporting a condition that would have been reported at an earlier wave, or misreporting the year of the disease onset. An encouraging result is that the reporting errors examined here were only marginally associated with socio-demographic predictors. This suggests that measurement errors in self-reported health may not be systematic in a way that would bias estimates of cancer disparities across social groups - rather, the errors appear fairly random across different groups. Our findings with regard to timing precision suggest that longitudinal analyses that rely on precise timing of the onset of disease to estimate long-term impacts of disease may be biased toward zero as a result of the inaccurate reports of the onset of disease [35]. Even for health events as salient as cancer, researchers should exercise caution about the presumed accuracy of cancer self-reports, especially if the timing of diagnosis is an important covariate.

## Competing interests

The authors declare that they have no competing interests.

## Authors' contributions

All authors contributed to the development of the research question, statistical analysis, and writing of the manuscript. AZ conducted the analysis and prepared the manuscript. JBD contributed to the initial planning of the study and helped significantly to draft and revise the manuscript. RFS contributed to the research idea, consulted on statistical analysis and interpretation of findings, and is a co-director and principal investigator of the source data. RBW contributed to the conception of the research question, assisted in revising the manuscript, and is a co-principal investigator of the source data. All authors have read and approved the final manuscript.
